# Ductus arteriosus banding to regulate excessive pulmonary blood flow in a neonate with necrotizing enterocolitis and complex congenital heart disease, including pulmonary atresia and total anomalous pulmonary venous return: a case report

**DOI:** 10.1186/s13019-022-02075-3

**Published:** 2022-12-20

**Authors:** Takayoshi Oyasu, Mineji Hayakawa, Noriyoshi Ebuoka, Junichi Oba

**Affiliations:** 1 Department of Emergency Medicine, Hokkaido University Hospital, Kita 15-Jo Nishi 7-Chome, Kita-Ku, Sapporo, Hokkaido 060-8638 Japan; 2 Department of Cardiovascular Surgery, Hokkaido Medical Center for Child Health and Rehabilitation, 1-1-240-6 Kanayama, Teine-Ku, Sapporo, Hokkaido 006-0041 Japan

**Keywords:** Ductus arteriosus banding, Necrotizing enterocolitis, Congenital heart disease, Right isomerism, Ductus-dependent pulmonary circulation, Case report

## Abstract

**Background:**

Patients with right isomerism have accompanying complex congenital heart disease, which is characterized by pulmonary atresia and total anomalous pulmonary venous return. Balanced regulation of the systemic and pulmonary circulation is essential for successful management, especially for cases complicated with necrotizing enterocolitis (NEC).

**Case presentation:**

A 6-day-old male neonate with a single ventricle, pulmonary atresia, patent ductus arteriosus (DA), and total anomalous pulmonary venous return associated with right isomerism was admitted because of dyspnea, cyanosis, and melena. The patient presented circulatory incompetence due to excessive pulmonary blood flow, resulting in NEC. The patient underwent DA banding and colectomy following continuous intravenous infusion of prostaglandin E1 at six days. Subsequently, his condition improved, reaching a systemic oxygen saturation of around 80%. He underwent a bidirectional Glenn procedure and closure of colectomy at the ages of 5 and 6 months, respectively.

**Conclusion:**

DA banding can be an alternative to placing an aortopulmonary shunt, which is conventional in patients with ductus-dependent pulmonary circulation, because DA banding is feasible without cardiopulmonary bypass.

## Background

Patients with right isomerism have accompanying complex congenital heart disease, characterized by pulmonary atresia and total anomalous pulmonary venous return. As the pulmonary circulation in these cases depends on a patent ductus arteriosus (DA), intravenous administration of prostaglandin E1 (PGE1) is required after birth. Achieving a balance between systemic and pulmonary circulation is essential for successfully managing these patients [1–3]. Meanwhile, necrotizing enterocolitis (NEC) is a devastating condition in neonates, especially preterm babies. NEC is believed to be responsible for intramural bacteria disrupting and invading intestinal epithelial cells with subsequent activation of an intestinal inflammatory response and decreased intestinal blood supply. NEC occasionally occurs in neonates with congenital heart disease [4, 5], with significant left-to-right shunting and/or cyanosis resulting in decreased diastolic gut perfusion pressures and reduced oxygenated blood flow decompensating the systemic circulation. The prevalence of NEC is reported to be 3.3–3.7% in newborn babies with congenital heart disease [4, 5]. We herein present the case of a neonate with ductus-dependent pulmonary circulation and NEC who underwent DA banding to regulate the pulmonary and intestinal blood flow.

## Case presentation

**Fig. 1:**
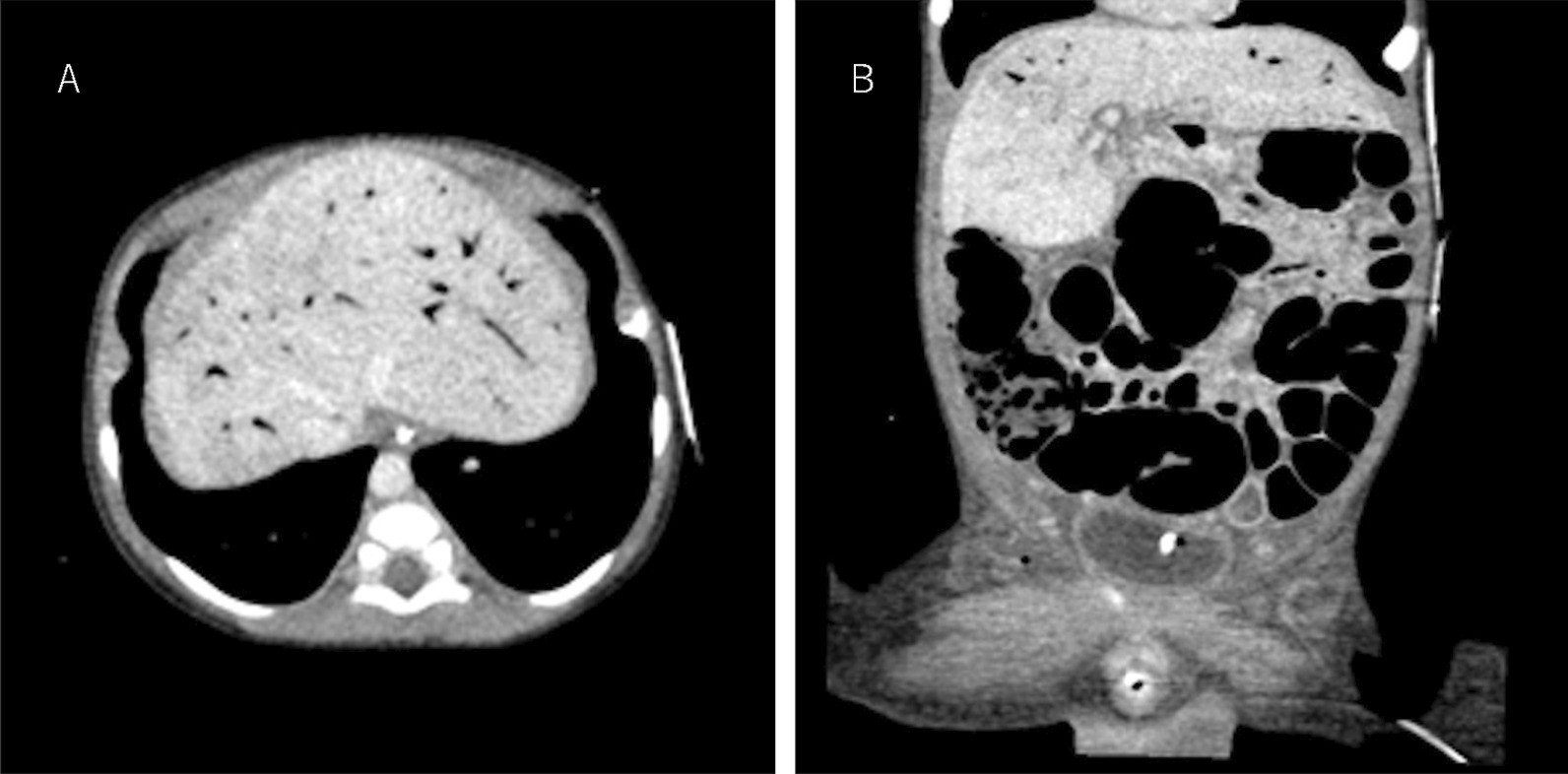
Contrast-enhanced computed tomography (CT) findings. **A** CT image depicting gas in the portal vein. **B** Extensive bowel dilatation and gas in the right colon wall without intra-abdominal free air are noted

A 6-year-old male neonate was referred to our hospital due to cyanosis, dyspnea, and melena. The patient was born at the gestational age of 39 weeks, weighing 2,690 g. Echocardiography after birth showed complete atrioventricular defect (Rastelli A), common atrium, pulmonary atresia, patent DA, bilateral superior vena cava, and cardiac-type total anomalous pulmonary venous return. His systemic oxygen saturation was around 90% after mechanical ventilation and intravenous administration of PGE1 (5.3 ng/kg/min). He presented worsening dyspnea because of excess pulmonary blood flow via a large DA. Next, we performed hypoxic therapy using nitrogen inhalation. At six days of age, progressing metabolic acidosis, systemic oxygen saturation over 93%, cyanosis, dyspnea, and melena were noted. Despite the intensive treatment, the patient presented circulatory incompetence, resulting in NEC. Based on computed tomography, NEC was confirmed by intestinal dilatation and portal vein gas accumulation (Fig. 1). Echocardiography following the visit showed moderate atrioventricular valve regurgitation but no pulmonary vein stenosis. Therefore, we attempted emergent surgery to regulate the systemic and pulmonary circulation by placing a Blalock-Taussig shunt.

### Surgical course

**Fig. 2:**
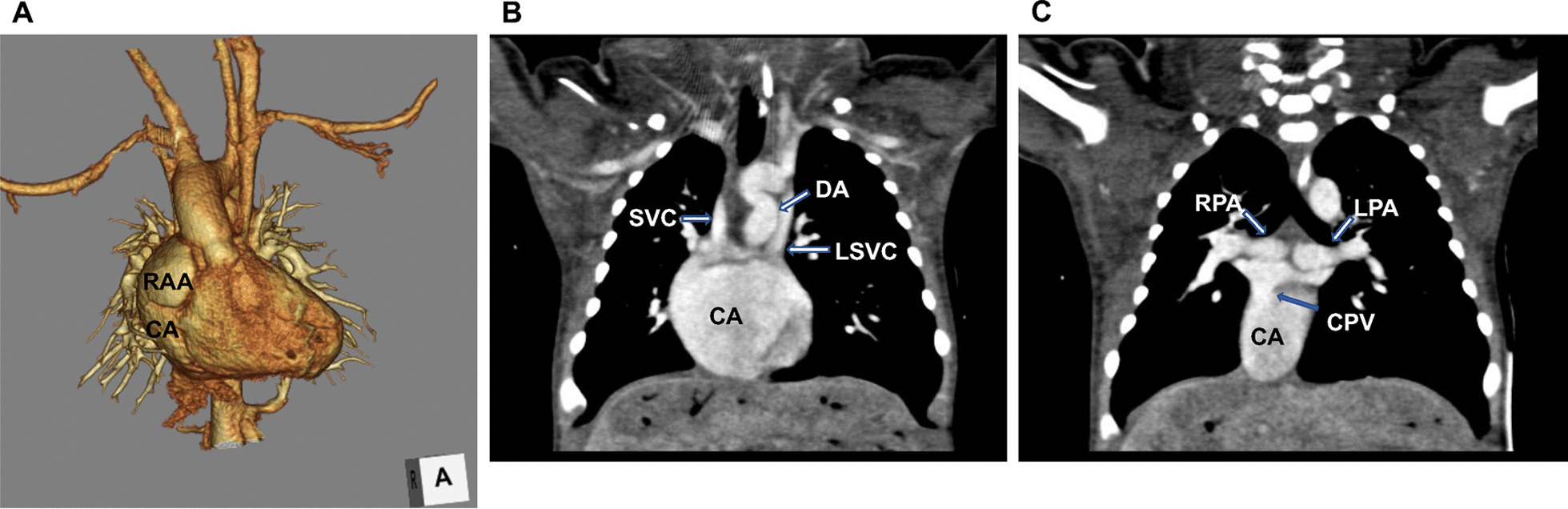
Preoperative contrast-enhanced computed tomography (CT) findings. **A** 3DCT image showing the right-sided juxtaposition of the atrial appendage overhanging the common atrium. **B** Coronal CT image showing the bilateral superior vena cava connected to the ceiling of the common atrium; large DA. **C** Coronal CT image showing CPV connected to the right-side common atrium. CA, common atrium; RAA, right atrial appendage; DA, ductus arteriosus; SVC, superior vena cava; LSVC, superior vena cava; RPA, right pulmonary artery; LPA left pulmonary artery; CPV, common pulmonary vein

We continued PGE1 due to the risk of DA closure during the operation. Following median sternotomy, his heart appeared in poor condition; atrial and ventricular tachycardia very easily developed whenever the heart was touched. We noted the pulmonary artery located deeply behind the common atrium (Fig. 2). This made placing a Blalock-Taussig shunt without cardiopulmonary bypass (CPB) hazardous and bilateral pulmonary artery banding difficult. Therefore, we undertook DA banding with a part of a 3-mm expanded polytetrafluoroethylene artificial graft. The banding circumference was 16 mm, which was determined to ensure a systemic oxygen saturation of around 80% and a partial arterial oxygen pressure above 40 mmHg at an inhaled oxygen fraction of 0.3–0.4. The chest remained open. Subsequently, colectomy and resection of a part of the small intestine were performed for NEC.

### Postoperative course

Intravenous administrations of adrenaline, antimicrobial agents, and PGE1 (2.5 ng/kg/min) were continued after admission to the intensive care unit. Serum lactate was 52 mg/dL (maximum, 400 mg/dL). In addition to systemic oxygen saturation and partial arterial oxygen pressure, we assessed the patient by daily echocardiography to evaluate the pulmonary artery, DA, and atrioventricular valve regurgitation to determine whether the band was adequate. His condition improved by the day. Delayed closure of the chest was performed on postoperative day 21. Enteral nutrition was started on postoperative day 27, and mechanical ventilation was discontinued on postoperative day 32.

PGE1 was continued to maintain pulmonary blood flow from the DA. Nutrition management was cautious due to the patient’s post-intestinal resection; therefore, he continued to receive inpatient care. At five months, he weighed 4.6 kg and had a systemic oxygen saturation of around 80%.

He underwent a bidirectional Glenn procedure, including the repair of total anomalous pulmonary venous return at five months and closure of colectomy at six months.

## Discussion and conclusions

The present case was notable because DA banding was performed to regulate the systemic and pulmonary circulation in a neonate with NEC and complex congenital heart disease, including pulmonary atresia and total anomalous pulmonary venous return. Usually, placing an aortopulmonary shunt is the conventional treatment for ductus-dependent pulmonary circulation. Bilateral pulmonary artery banding is one of the choices for regulating pulmonary flow without CPB in patients under such poor conditions. However, these procedures are problematic if the pulmonary artery is not adequately secured. Recent reports have shown that DA banding can be an alternative to regulate pulmonary circulation in patients with circulatory incompetence or complex anatomical issues [6–8].

DA banding has several advantages. First, it is a more straightforward and less invasive procedure than placing an aortopulmonary shunt. It is tolerated in the emergent setting requiring cardiopulmonary resuscitation. Second, anticoagulation is unnecessary during operation, which is a relevant advantage as compromised patients occasionally present a bleeding diathesis or disseminated intravascular coagulopathy.

A disadvantage of DA banding is that the pulmonary blood flow after DA banding tends to be more unstable than that after placing an aortopulmonary shunt. In addition, intravenous infusion of PGE1 is required to maintain pulmonary circulation despite DA banding. Furthermore, there are no established criteria for identifying the optimal circumferential length around the DA in these cases [9, 10], although the general criteria for pulmonary banding have been established by Trusler and Mustard [11] and Albus et al. [12]. Earle et al. [9] described that the target oxygen saturation was 60–70% when performing ductus banding. Matsumoto et al. [10] described that a partial arterial oxygen pressure should be kept above 50 mmHg. According to these reports, we successfully determined the circumferential length based on both systemic oxygen saturation and partial arterial oxygen pressure.

In conclusion, we present the case of a neonate with ductus-dependent pulmonary circulation and NEC who underwent DA banding to regulate pulmonary and intestinal blood flow. DA banding can be an alternative to the conventional placement of an aortopulmonary shunt in patients with ductus-dependent pulmonary circulation because DA banding is feasible without cardiopulmonary bypass.

## Data Availability

Data sharing is not applicable to this article as no datasets were generated or analyzed during the current study.

## Abbreviations

CPB: Cardiopulmonary bypass
DA: Ductus arteriosus
NEC: Necrotizing enterocolitis
PGE1: Prostaglandin E1
